# Green Nanoemulsion Stabilized by In Situ Self-Assembled Natural Oil/Native Cyclodextrin Complexes: An Eco-Friendly Approach for Enhancing Anticancer Activity of Costunolide against Lung Cancer Cells

**DOI:** 10.3390/pharmaceutics14020227

**Published:** 2022-01-19

**Authors:** Nabil A. Alhakamy, Shaimaa M. Badr-Eldin, Osama A. A. Ahmed, Hibah M. Aldawsari, Solomon Z. Okbazghi, Mohamed A. Alfaleh, Wesam H. Abdulaal, Thikryat Neamatallah, Omar D. Al-hejaili, Usama A. Fahmy

**Affiliations:** 1 Department of Pharmaceutics, Faculty of Pharmacy, King Abdulaziz University, Jeddah 21589, Saudi Arabia; nalhakamy@kau.edu.sa (N.A.A.); oaahmed@kau.edu.sa (O.A.A.A.); haldosari@kau.edu.sa (H.M.A.); maalfaleh@kau.edu.sa (M.A.A.); al-shaery111@hotmail.com (O.D.A.-h.); uahmedkauedu.sa@kau.edu.sa (U.A.F.); 2 Center of Excellence for Drug Research and Pharmaceutical Industries, King Abdulaziz University, Jeddah 21589, Saudi Arabia; 3 Department of Pharmaceutics and Industrial Pharmacy, Cairo University, Cairo 11562, Egypt; 4 Global Analytical and Pharmaceutical Development, Alexion Pharmaceuticals, New Haven, CT 06510, USA; sokbazghi@gmail.com; 5 Vaccines and Immunotherapy Unit, King Fahd Medical Research Center, King Abdulaziz University, Jeddah 21589, Saudi Arabia; 6 Department of Biochemistry, Faculty of Science, Cancer and Mutagenesis Unit, King Fahd Medical Research Center, King Abdulaziz University, Jeddah 21589, Saudi Arabia; whabdulaal@kau.edu.sa; 7 Department of Pharmacology and Toxicology, Faculty of Pharmacy, King Abdulaziz University, Jeddah 21589, Saudi Arabia; taneamatallah@kau.edu.sa

**Keywords:** ecofriendly, nanoparticles, lung cancer, venoms, cytotoxicity

## Abstract

Lung cancer is the second-most deadly malignancy worldwide, of which smoking is considered a major risk factor and causes 75–80% of lung cancer-related deaths. Costunolide (CTD) extracted from plant species *Saussurea, Aucklandia, and Inula* exhibits potent anticancer properties, specifically in lung cancer and leukemia. Several nanoemulsions were prepared and optimized using a three-factor Box–Behnken experimental design. The optimized green nanoemulsion (GNE) showed a vesicle size of 199.56 nm. The IC_50_ values revealed that A549 cells were significantly more sensitive to the optimized CTD formula than the plain formula and raw CTD. A cell cycle analysis revealed that the optimized CTD formula treatment resulted in significant cell cycle arrest at the S phase. The results also indicated that treatment with the CTD formula significantly increased caspase-3, Bax, Bcl-2, and p53 mRNA expression compared to the plain formula and CTD raw. In terms of the inflammatory markers, the optimized formula significantly reduced the activity of TNF-α and NF-κB in comparison with the plain formula and raw drug only. Overall, the findings from the study proved that a CTD GNE formulation could be a promising therapeutic approach for the treatment of lung cancer.

## 1. Introduction

According to the World Health Organization’s (WHO) estimated top 20 causes of death in 2016, cancer was listed as the first [1]. Among many cancers, lung cancer is the main cause of cancer-related death among men, especially in developed countries, and it is becoming more common among women [2]. The incidence of lung cancer is very high not only in United State but also in Canada, China, and many other industrial countries [3]. In 2008, 1.6 million new cases of lung cancer were reported in 91 countries around world, and 1.4 million deaths were documented as a result of lung cancer in 17 countries [4]. Although many environmental and lifestyle factors lead to developing lung cancer, cigarette smoking habits are strongly attached to the incidence of lung cancer and its mortality rate [5,6]. In fact, smoking is the highest contributor factor and represents 85–95% of the lung cancer epidemiology [6]. According to the WHO, lung cancer is classified as non-small cell lung cancer (NSCLC), which represents 85%, and small cell lung cancer (SCLC), which represents 15%. The main common subtypes of NSCLC are lung adenocarcinoma and lung squamous cell carcinoma [7].

Chemotherapy and radiation therapy are the standard treatments for patients with lung cancer [8]. Although chemotherapy can suppress the symptoms of lung cancer and plays an important role in enhancing the patient’s quality of life, it does not prolong the patient’s survival [9]. Furthermore, chemotherapy is associated with many side effects, such as nausea, vomiting, fatigue, cardiac complication, anemia, and neutropenia [10]. On the other hand, chemotherapy-acquired resistance has been demonstrated in non-small cell lung cancer cell lines [11]. As a result, improved treatment alternatives for lung cancer are critical. For thousands of years, medicinal plants have been utilized to cure cancer in ancient Egypt, India, China, and the Arab world. According to the statistics, over 3000 plant species have been utilized to cure cancer across the world. Anticancer agents derived from plants are a valuable source of anticancer drugs, because they have more structurally varied “drug-like” and “biologically friendly” molecular properties compared to pure synthesized molecules at random [12]. In Chinese herbal medicines, especially, phytochemicals have shown promise as a therapeutic option for cancer [13]. Phytochemical costunolide (CTD) is a member of a large family of 5000 compounds of sesquiterpene lactones that has multiple pharmacological activity [14]. CTD has been isolated from a variety of medicinal plant species, including *Costus speciosus, Saussurea lappa,* and *Laurus nobilis* [15]. It has been reported to possess antiviral, antifungal, anti-inflammatory, antiulcer, antioxidant, antidiabetic, and antitumor activity [14,15]. In lung cancer, CTD has been shown to inhibit the proliferation of cell-induced apoptosis and prevent angiogenesis. The molecular mechanism through which CTD suppresses tumor metastasis is still unclear [8].

In the research of cancer therapy, scientists have focused more on nanoemulsion due to its essential properties that achieve effective therapeutic goals through providing a large surface area, as well as increasing the drug’s half-life, with selectivity in targeting [16]. Nanoemulsion as a carrier for a lipophilic compound (CTD) is a colloidal system consisting of two immiscible liquids stabilized by emulsifiers [17]. In the literature, primarily, two techniques for the formulation of nanoemulsions have been documented. The first is a high-energy emulsification approach, while the second is a low-energy method [18]. In the low-energy method for emulsion preparation, large amounts of stabilizer (surfactant) are required, and this is unfortunately harmful and induces inherent toxicity [19]. According to the concepts of green engineering and green chemistry, the pharmaceutical approach has recently focused on minimizing and/or replacing the use of synthetic surfactants and organic solvents in preparations of drug delivery systems [20]. In our work, biosurfactant α-cyclodextrin (α-CD) was utilized to prepare the green nanoemulsion (GNE), which has ecofriendly properties with less adverse effects on humans. α-CD as a macrocyclic oligosaccharide consists of six glucose units and is biodegradable, biocompatible, and nontoxic to humans [20,21]. Since it has a hydrophobic core cavity and a hydrophilic outer surface, α-CD is capable of forming inclusion complexes with different molecules and, thus, will stabilize the nanoemulsion through a self-assembled complex with fatty acids at the oil–water interface [20]. As a result, the self-assembled natural oil/native cyclodextrin complex acts as a carrier for the included CTD in an aqueous environment and thus improves the solubility and rate of dissolution, resulting in enhancement of the bioavailability.

## 2. Materials and Methods

### 2.1. Materials

CTD, α-CD (MW = 972 Da) and pumpkin oil were purchased from Sigma-Aldrich (GmbH, Steinheim am Albuch, Germany, Baden-Württemberg, Germany). All other chemicals and solvents were of analytical grade.

### 2.2. Experimental Design for Formulation and Optimization of CTD-Loaded GNE

A response surface experimental design, namely the Box–Behnken, was employed for the development and optimization of CTD-loaded GNE. Three numerical factors, namely pumpkin oil concentration (X_1_, %), CD concentration (X_2_, %), and homogenization time (X_3_, min), were considered as independent variables, whereas the globule size (Y, nm) was studied as the response. The used ranges were selected based on preliminary studies. Design Expert^®^ software (Version 12.0, Stat-Ease Inc., Minneapolis, MN, USA) was employed to generate the design points and statistically analyze the response data. The design resulted in 15 experimental runs, including three replicate center points. The globule size was evaluated using linear, 2-factor interactions, and quadric sequential models. The model with highest prediction power was selected to assess the impact of the studied variables on the claimed response. Analysis of variance (ANOVA) was performed to test the statistical significance of the independent variables and the interactions between them at the 95% level. The composition of the optimized emulsion with a minimized globule size was predicted using numerical optimization following the desirability approach. The selected optimized emulsion was subjected to further testing. The variables’ levels and the criterion set for the optimized formulation are summarized in Table 1. The variables’ levels in each experimental run with the corresponding globule size are displayed in Table 2.

### 2.3. Preparation of CTD-Loaded GNE

A reported method based on high shear homogenization with modifications was utilized to prepare CTD-loaded GNE [22]. A specified amount of CTD, α-CD, limonene oil, and water were placed in glass bottles and homogenized for 3 min at 25 °C (T25 digital Ultra-turrax^®^, IKA, Staufen, Germany) operating at 20,000 rpm. Then, deionized water was poured into the mixture. The coarse emulsion was homogenized at 20,000 rpm for different time intervals, as indicated in Table 1. The quantities used in the prepared emulsions were specified according to the compositions of the experimental design runs.

### 2.4. Characterization of CTD-Loaded GNE

#### 2.4.1. Globule Size

The dynamic light scattering technique was applied for the determination of the globule sizes of the prepared emulsions using Zetasizer Nano ZSP (Malvern Panalytical Ltd., Malvern, UK). Before measurements, the prepared emulsions were appropriately diluted with double-distilled water. Each measurement was done thrice, and the results were presented as the mean ± standard deviation.

#### 2.4.2. Transmission Electron Microscope (TEM)

The appropriately diluted optimized CTD-loaded GNE was investigated using a transmission electron microscope (Jeol JEM1230, Tokyo, Japan). A drop of the formula was negatively stained with 1% phosphotungstic acid on a copper grid. The samples were then examined and photographed under the microscope after being dried at ambient temperature for 15 min.

### 2.5. In Vitro Anticancer Activity of Optimized CTD-Loaded GNE in A549 Cells

#### 2.5.1. Determination of IC_50_ by MTT Assay

The IC_50_ values of untreated A549 cells (control) or treated with blank GNE, CTD, or CTD-GNE for 24 h were obtained using the MTT (3-(4,5-dimethylthiazol-2-yl)-2,5-diphenyltetrazolium bromide) viability assay, as previously described [23]. Briefly, A549 cells (1 × 10^5^ cells) were seeded into a 96-well plate and incubated overnight for complete attachment. Later, the cells were treated with the blank GNE, CTD, or CTD-GNE at 0.39 µM, 1.56 uM, 6.26 uM, 25 uM, and 100 uM. After 24 h, the medium was replaced with MTT solution (2 mg/mL), and the plates were incubated at 37 °C for 4 h. The purple formazan product was dissolved by the addition of 200 µL of 100% DMSO, and the plates were then incubated for 5 min at 37 °C in a 5% CO_2_ incubator. The absorbance at 569 nm in each well was read by using a microplate reader (Spark^®^ multimode, Tecan Group Ltd., Seestrasse, Maennedorf, Switzerland). The results were expressed as the percent of cell viability relative to the control. Dose response curves were plotted and IC_50_ values. The IC_50_ for each of the experimental conditions was calculated using GraphPad prism software (GraphPad, Inc., La Jolla, CA, USA).

#### 2.5.2. Cell Cycle Analysis

The analysis of cell cycle stages in the A549 cells was performed by flow cytometry (FACScalibur, BD Bioscience, San Diego, SD, USA), as previously described [24]. Briefly, cells were seeded in 6-well plates at a density of 3 × 10^5^ cells/well and left untreated (control) or treated with blank GNE, CTD, or CTD-GNE at a subtoxic concentrations (IC_10_) for 24 h. Afterthought, the cells were washed, fixed with 70% cold ethanol, and stained with propidium iodide and RNase staining buffer. A total of 10,000 gated events were acquired using flow cytometry (FACS Calibur, BD Bioscience, San Diego, SD, USA) in order to assess the cell cycle, and the data were analyzed by MultiCycle AV software (Phoenix Flow Systems, San Diego, CA, USA).

#### 2.5.3. Annexin V–FITC Apoptosis Assay

The apoptotic effects with blank GNE, CTD, or CTD-GNE A549 cells were assayed using an Annexin V-FITC Apoptosis Detection Kit (BioVision, Cambridge BioSciences, Cambridge, UK) according to the manufacturer’s protocols. Briefly, cells were seeded into 6-well plates at a density of 1 × 10^6^ cells/well and were left untreated (control) or treated with the plain, CTD-R, or Optimized CTD formula at a subtoxic concentrations (IC_10_) for 24 h. Next, the cells were centrifuged, separated, washed with PBS buffer, and, finally, resuspended in 500 μL of 1× binding buffer. The Annexin-V FACS analyses were performed using a Cytek^®^ Northern Lights 2000 spectral flow cytometer (Cytek Biosciences, Fremont, CA, USA) and using SpectroFlo™ Software version 2.2.0.3 (Cytek Biosciences, Fremont, CA, USA).

#### 2.5.4. Real-Time Polymerase Chain Reaction (RT-qPCR)

##### RNA Extraction

RNA was extracted from A594 cells using Qiagen’s RNeasy Mini Kit (Qiagen, Manchester, UK) according to the manufacturer’s instructions. The concentration and purity of RNA was confirmed using a Nanodrop spectrophotometer (ND-2000C, Thermo Fisher Scientific, Waltham, MA USA). A ratio of A260 nm/A230 nm of no less than 1.8 and A260 nm/A280 nm ratio of no less than 1.9 were detected in all RNA samples.

##### cDNA Synthesis and PCR Amplification

RNA was normalized between tubes and reverse-transcribed to complementary DNA (cDNA) using the iScript™ One-Step RT-PCR Kit with the SYBR^®^ Green Kit (Bio-Rad, Hercules, CA, USA) according to the manufacturer’s manual. Relative expression patterns of p53, bcl2, casp3, and β-Actin were performed using 10 ng of RNA template in a 50-mL reaction mixture of the iScript One-Step RT-PCR Kit with SYBR^®^ Green Mix using a 7500 Fast Real-Time PCR System (Applied Biosystems, Thermo Fisher Scientific Corporation, Waltham, MA, USA).

#### 2.5.5. Statistical Analysis

GraphPad Prism software (GraphPad Software, San Diego, CA, USA) was utilized for the statistical analysis. The data were presented as the mean ± SD. The means were compared by using the analysis of variance (ANOVA), which was then followed by Tukey’s post hoc test. Statistical significance was indicated at *p* < 0.05.

## 3. Results

### 3.1. Experimental Design

#### 3.1.1. Fit Statistics and Diagnostic Analysis

**Figure 1:**
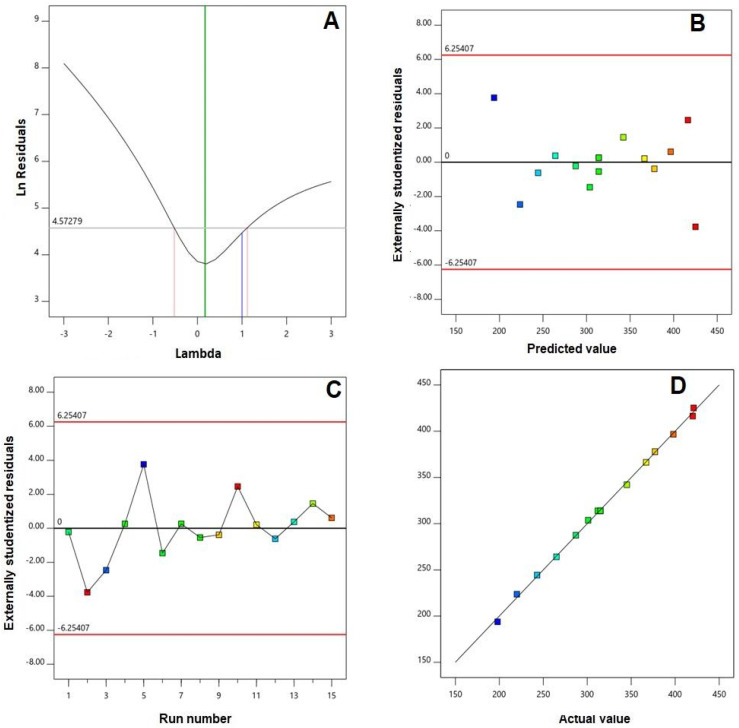
Diagnostic plots for the particle sizes of CTD GNE. (**A**) Box-Cox plot for power transforms, (**B**) externally studentized residuals vs. predicted values, (**C**) externally studentized residuals vs. run number, and (**D**) predicted vs. actual values plot.

A fit statistics summary for the globule sizes is compiled in Table 3. Amongst the polynomial models (linear, two-factor interaction, and quadratic) under investigation, the quadratic model was the best fitting model for the globule sizes of CTD-loaded GNE, as evidenced by its greatest R^2^ and least PRESS values. The adjusted R^2^ was in good harmony with the predicted R^2^. Additionally, the adequate precision value of 67.80 exceeding the preferable value implies an adequate signal-to-noise ratio. Thus, the quadratic model could be regarded as successfully exemplary to explore the experimental design space. Diagnostic plots for the globule size, developed for establishing the goodness of fit of the selected model, are displayed in Figure 1. The Box–Cox plot for power transforms, Figure 1A, exhibits a recommended lambda (λ) value of 0.17. The 95% confidence interval (shown by the red limits) includes the current λ value of 1; therefore, no specific transformation for the measured globule size is suggested [25,26]. The absence of a need for transformation is advocated by the calculated maximum-to-minimum size ratio of 1.97, where a ratio greater than 10 probably necessitates transformation. Random distribution of the measured globule sizes in the externally studentized residuals vs. predicted response plots, Figure 1B, within the limits indicates that no constant error exists. Moreover, the residual vs. run plots, Figure 1C, display random point distribution, indicating that no lurking factor could have an impact on the measured sizes. The predicted vs. observed size plots, Figure 1D, show a remarkably linear correlation that highlights the good agreement between the observed and predicted values, which further confirms the reliability of the selected model [27].

#### 3.1.2. Variables’ Impact on Globule Size (Y)

The globule size is an influential criterion when developing an emulsion, owing to its impact on biopharmaceutical drugs [20]. In addition, it is reported that globule sizes of lipid-based delivery systems of less than 400 nm favor preferential distribution within solid cancerous tissues [28,29]. Therefore, the prepared CTD-loaded GNE exhibited acceptable globule sizes that ranged from 208 ± 6.5 to 411 ± 12.9 nm. However, the favored buildup of nanodelivery systems and the associated clinical efficacy could be prevailed upon by ineffective tumor penetration owing to the tumor pathological characteristics [30]. Cancerous cells penetration could be boosted by reducing the globule size to enlarge the surface area available for permeation. Accordingly, the globule size of the GNE was optimized to the minimized value to assure effective tumor penetration. The analysis of variance (ANOVA), according to the selected quadratic model, showed a F-value of 430.85 (*p* < 0.0001). As per the coded factor, the quadratic model equation for the globule size was generated as follows:

Y = 314.00 + 76.25 X_1_ − 29.38 X_2_ − 10.13 X_3_ + 5.75 X_1_X_2_ + 0.25 X_1_X_3_ + 2.00 X_2_X_3_ − 1.75 X_1_^2^ + 3.00 X_2_^2^ + 8.00 X_3_^2^

**Figure 2:**
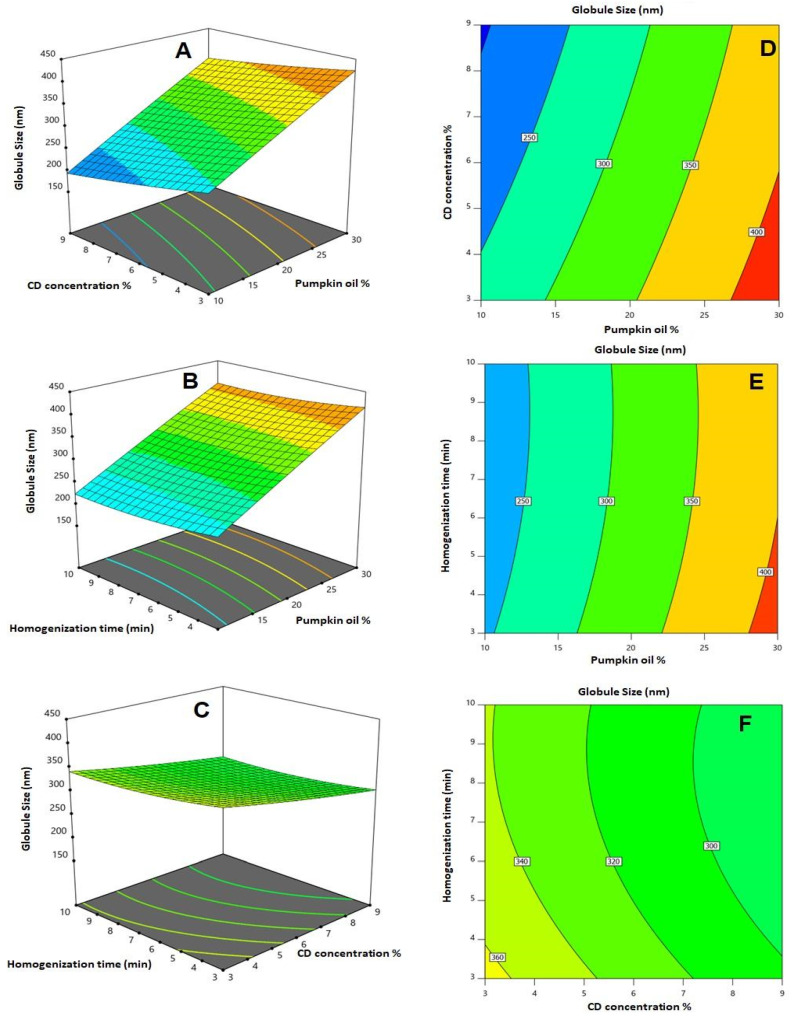
2D contour plots (**A**–**C**) and 3D surface plots (**D**–**F**) for the influence and interactions of pumpkin oil concentration (X_1_), α-CD concentration (X_2_), and homogenization time (X_3_) on the globule size of CTD-loaded GNE.

All linear terms (X_1_, X_2_, and X_3_) expressing the independent variables showed a significant impact on the globule size (*p* < 0.0001 for X_1_ and X_2_, *p* = 0.01 for X_3_). The interaction terms between pumpkin oil and CD concentrations (X_1_ and X_2_), in addition to the quadratic term X_3_^2^ corresponding to the homogenization time, were also significant (*p* = 0.0402 and 0.0143, respectively). The effects and the interactions between the investigated variables on the globule size are illustrated as 2D contour plots and 3D surface plots in Figure 2. It can be seen that the globule size increases with the increasing pumpkin oil concentration, while it decreases with the increasing CD concentration. This could be assisted by the signs of the coefficients in the model equation. The positive sign of the oil concentration reflects the direct relationship with the globule size, while the negative sign of both the CD concentration and homogenization time reflects the inverse relationship between these factors and the predicted response. On the other hand, the magnitude of the coefficient reflects the magnitude of the variable impact on the globule size. As per the quadratic model equation, the order of influence of the variables on the size is the oil concentration > CD concentration > homogenization time, as depicted by the order of magnitude of the coefficients of the linear terms X_1_, X_2_, and X_3_, respectively.

#### 3.1.3. Optimization

The numerical optimization method was selected according to the constraints previously set on the globule size; the optimized levels of the investigated variables were anticipated as follows: 10.1% for the pumpkin oil concentration, 8.8% for the α-CD concentration, and 6.2 min for the homogenization time. The globule size of the optimized formulation was evaluated. The percentage error (1.12%) between the predicted (197.34 nm) and obtained particle sizes (199.56 nm) was relatively low, highlighting the appropriateness of the optimization process.

### 3.2. Transmission Electron Microscope (TEM)

**Figure 3:**
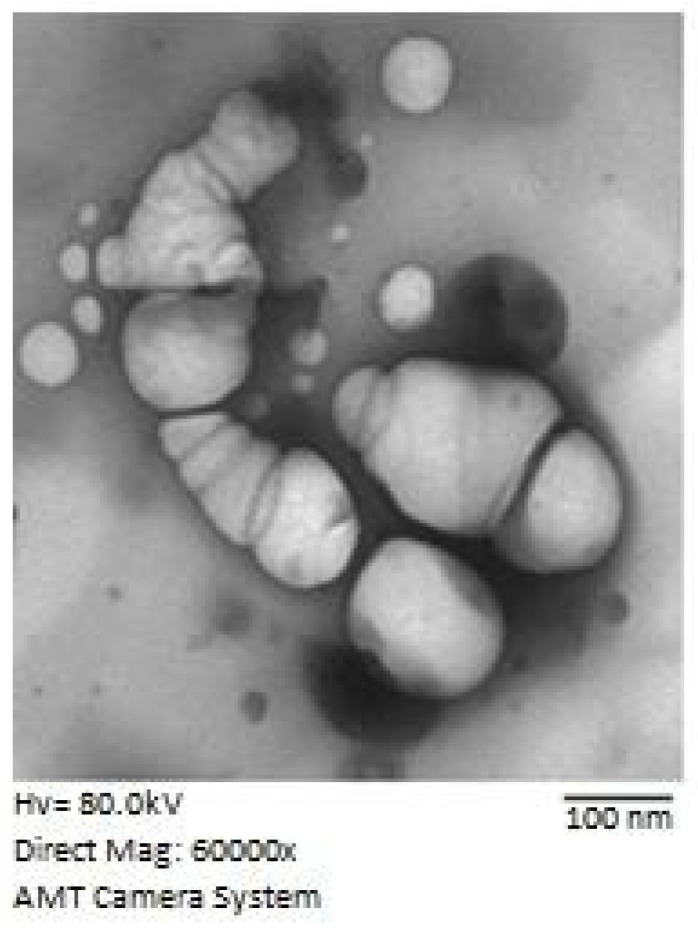
TEM image of the optimized CTD-GNE formula.

TEM images of the optimized CTD-GNE formula showed spherical-formed structures (Figure 3). The sizes of the spherical structures showed relatively similar average sizes as the GNE sizes obtained by the dynamic light scattering technique.

### 3.3. Cytotoxicity Assay

**Figure 4:**
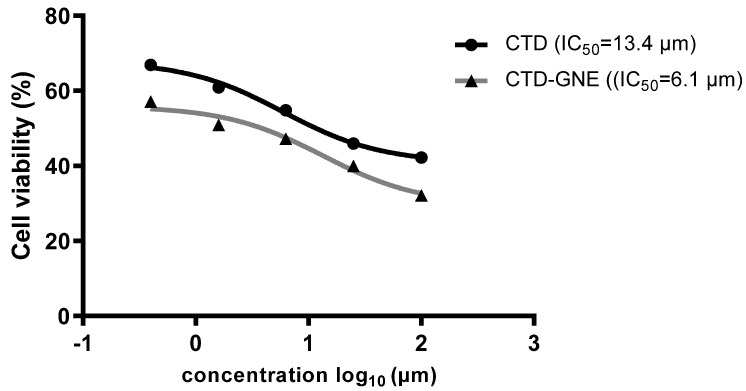
Viability of A549 cells using the MTT assay after 24 h of incubation with CTD or CTD-GNE with IC_50_ calculations.

A549 cell viability by CTD or CTD-GNE was assessed via the MTT assay. Inhibition occurred in a dose-dependent manner. CTD was demonstrated to inhibit the cells’ viability, with an IC_50_ value of 13.4 ± 1.5 µM (Figure 4). CTD-GNE-treated cells showed a significant reduction in their viability compared to the CTD group (*p* < 0.05). CTD-GNE exhibited an IC_50_ value of 6.1 ± 0.8 µM. An IC_50_ value of ~24 μM was previously reported with CTD against A549 cells. Based on these values, our data suggest that the CTD-GNE could easily penetrate the cell membrane and induce cell death at a significantly low dose.

### 3.4. Cell Cycle Analysis

**Figure 5:**
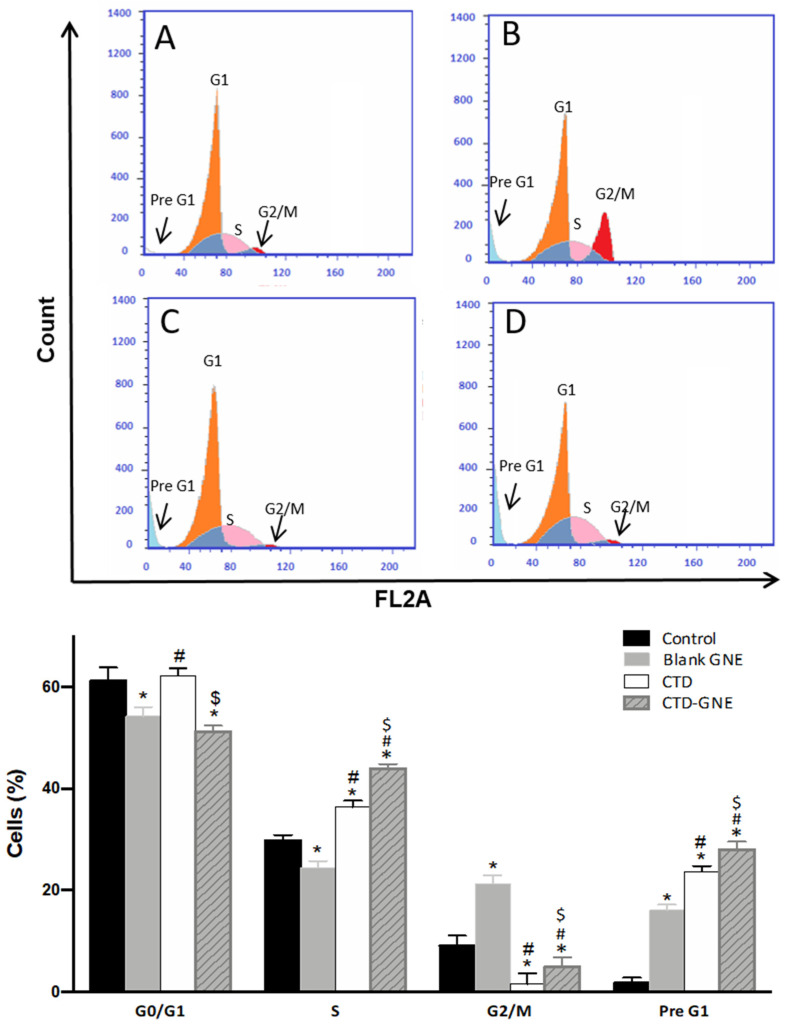
Graphical presentation of the A549 cell cycle phases in (**A**) control, (**B**) blank GNE, (**C**) CTD, (**D**) CTD-GNE using a flow cytometry analysis. All data are expressed as the mean ± SE of three independent experiments. * *p* < 0.05 is considered significantly different from the control, ^#^ *p* < 0.05 is considered significantly different from blank GNE, and ^$^ *p* < 0.05 is considered significantly different from CTD.

To determine whether CTD-GNE inhibited the viability of the A549 cells through influencing the cycle phases, we treated the cells with blank, CTD, or CTD-GNE for 24 h and analyzed the cell cycle using flow cytometry. As presented in Figure 5, the control A549 cells exhibited a proliferative profile of about 60% at the G0/G1 phase, 25% at the S phase, 9% at the G2 phase and 1.7% at the pre-G1 phase. The CTD treatment significantly increased the number of cells in the cell cycle’s G1 and S phases in comparison to the control and blank GNE (*p* < 0.05). The percentage of cells in G2/M decreased to 1.5 ± 2.1%, indicating a cell cycle arrest at the S phase. In addition, the percentage of cells increased in the pre-G1 phase to 23.6 ± 1.1% when compared to the control cells, and the appearance of a clear sub-G1 peak represented cell death. Interestingly, CTD-GNE significantly increased the percentage of G2/M when compared to CTD, indicating a rapid G2/M arresting activity (*p* < 0.05). A slight incline of the G0/G1 phase accompanied this, as did a marked increase of the S phase and pre-G1 phase, which suggest a significant arrest of the A549 S phase, along with a significant apoptotic effect, in comparison to CTD alone (Figure 5).

### 3.5. Annexin V Apoptosis Assay

**Figure 6:**
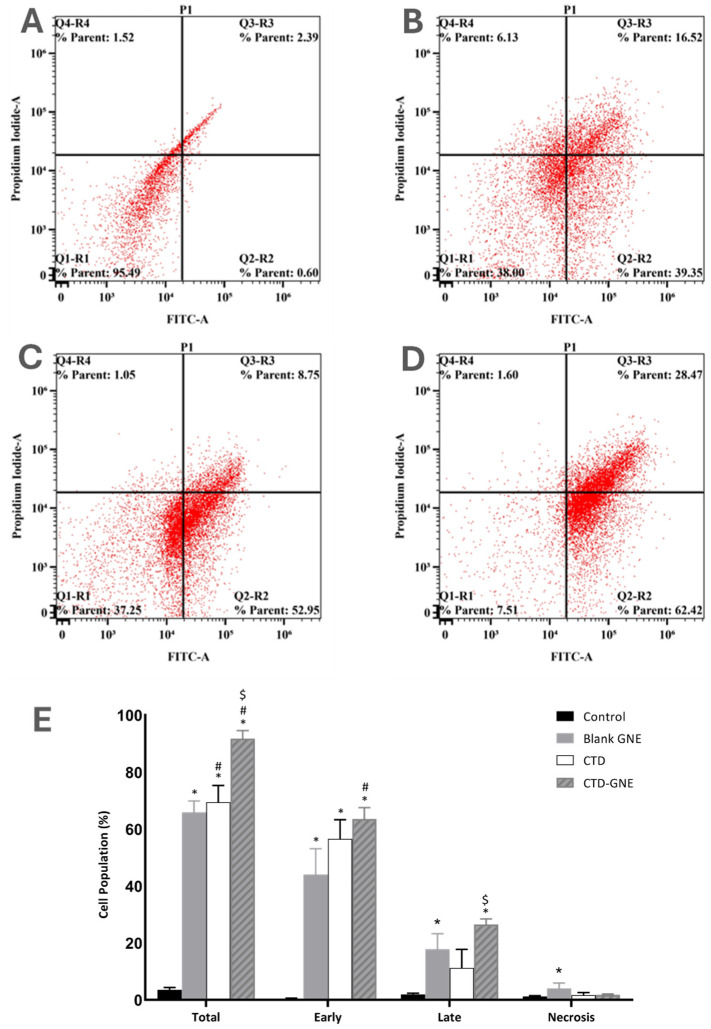
Analysis of apoptosis using the Annexin-V staining in A549 cells: (**A**) the control, (**B**) blank GNE, (**C**) CTD, (**D**) CTD-GNE and (**E**) the percentage of apoptotic or necrotic cells using flow cytometric analysis. All data are expressed as the mean ± SE of three independent experiments. * *p* < 0.05 is considered significantly different from the control, # *p* < 0.05 is considered significantly different from the blank GNE, and $ *p* < 0.05 is considered significantly different from CTD.

To confirm whether the significant antiproliferative effect of CTD-GNE against A549 cells was mediated via apoptosis, the cells were treated with blank GNE, CTD, or CTD-GNE for 24 h. The apoptotic rate was investigated via flow cytometry based on Annexin V and PI staining. CTD-GNE significantly increased the percentage of early, late, and total apoptosis in A549 cells compared to the control, blank GNE, and CTD-treated cells (Figure 6). CTD at 10 µM has previously induced a similar apoptotic effect when A549 were treated for 24 h [8]. The apoptotic effect of CTD against human lung cancer cell line H1299 has been also previously reported. CTD could only induce a comparable apoptotic effect at 48 µM [8]. Our data demonstrated that CTD-GNE induced a significant apoptotic effect on A549 cells at a very low dose (IC_10_). Changes in the A549 apoptotic profile indicated that the cell underwent apoptosis due to CTD-GNE-inducing antitumor activity. The CTD-GNE possible apoptotic mechanism will be further investigated though its effect on caspase-3 and p53 expressions.

### 3.6. Caspase-3 Expression

**Figure 7:**
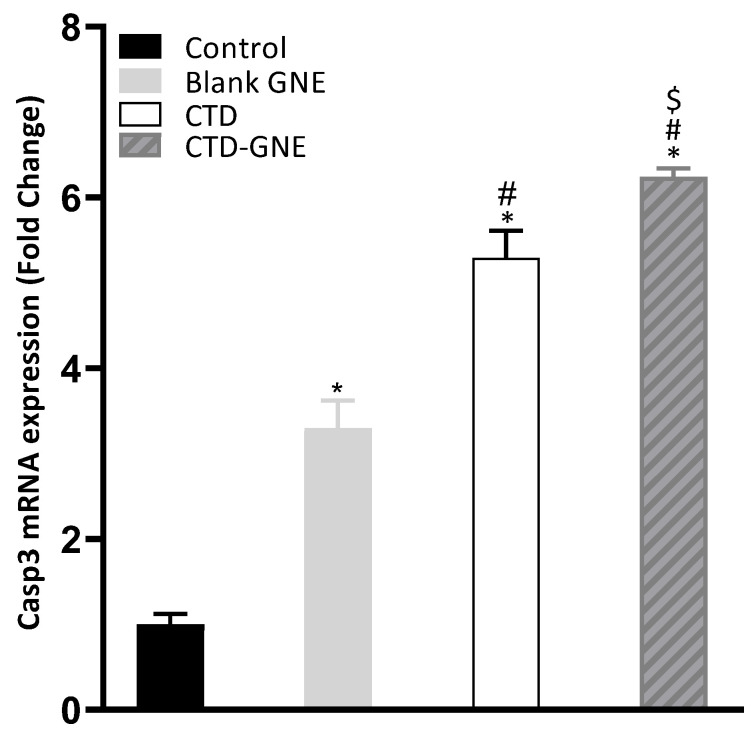
RT-qPCR analysis of the *casp3* mRNA expression levels. Expression levels were normalized to the reference gene *β*-actin using the comparative Ct method (2^−ΔΔCt^). All data are expressed as the mean ± SE of three independent experiments. * *p* < 0.05 is considered significantly different from the control, ^#^ *p* < 0.05 is considered significantly different from blank GNE, and ^$^ *p* < 0.05 is considered significantly different from CTD.

Caspases are a large family of cysteine proteases that are essential for initiating and executing the intrinsic apoptosis pathway. To determine whether the caspase was involved in CTD-GNE-induced apoptosis, we examined the expression of caspase-3, which is a common downstream apoptosis effector [31]. We examined the activation of *casp3* mRNA by RT-qPCR for blank GNE, CTD, and CTD-GNE. The A549 cells showed a significant upregulation of *casp3* expression in CTD-GNE when compared to CTD, while both blank GNE and CTD-treated cells showed an early induction of *casp3* (Figure 7). In a previous study, CTD increased caspase-3 with at least a dose of 10 μM compared to the control cells. Our results clearly demonstrated that the intrinsic-mediated caspase-3 activation pathway is involved in CTD-GNE-mediated apoptosis at a significantly low dose.

### 3.7. P53 Expression

**Figure 8:**
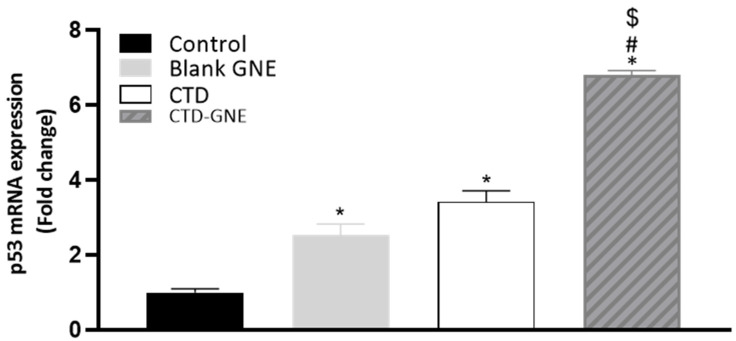
Effect of blank GNE, CTD raw, or CTD-GNE treatments on p53 mRNA expression in A549 cells. All data are expressed as the mean ± SE of three independent experiments. * *p* < 0.05 is considered significantly different from the control, ^#^ *p* < 0.05 is considered significantly different from blank GNE, and ^$^ *p* < 0.05 is considered significantly different from CTD.

The p53 gene is a tumor suppressor gene, which is upregulated rapidly in response to potentially oncogenic stimuli. A p53-mutated gene decreases the activity of the p53 protein, which leads to uncontrolled cell division [32]. The RT-qPCR results in Figure 8 indicate that the mRNA expression levels of p53 were significantly higher in CTD-GNE compared with the control, blank GNE, and CTD (*p* < 0.05). In a previous work, the same cells were treated with different doses of CTD (40 and 80 µM) for 24 h to induce p53 expression [9]. Both doses were significantly higher than CTD-GNE. Our results demonstrated that p53 played a critical role in CTD-GNE-mediated apoptosis at a very low dose in the A549 cell line.

## 4. Conclusions

The Box–Behnken design was successfully applied to optimize CTD-GNE; the optimized formulation prepared at 10.1% for the pumpkin oil concentration, 8.8% for the α-CD concentration, and 6.2 min for the homogenization time showed a minimized globule size of 199.56 nm. The low percentage of less than 5% between the predicted and measured sizes highlighted the appropriateness of the optimization process. The optimized formulation showed an enhanced cytotoxic and proapoptotic effect in lung cancer cells. Further, the optimized formula significantly reduced the activity of inflammatory markers in comparison with the plain formula and raw drug only. Overall, the findings from this work indicated that the proposed formulation could be a promising therapeutic approach for treating lung cancer.

## Figures and Tables

**Table 1:** Independent variables’ levels and desirability constraints for the globule sizes used in Box–Behnken for the optimization of CTD-loaded GNE.

Variables	Levels	
	(−1)	(+1)
A: Pumpkin oil concentration (%)	10	30
B: α-CD concentration (%)	3	9
C: Homogenization time (min)	3	10
**Response**	**Desirability Constraint**	
Globule size (GS, nm)	Minimize	

**Table 2:** Box–Behnken experimental runs of CTD-loaded GNE and their corresponding globule sizes.

Run	Independent Variables			Response
	X_1_: Pumpkin Oil Concentration (%)	X_2_: α-CD Concentration (%)	X_3_: Homogenization Time (min)	Y: Globule Size ± SD (nm) *
E1	20.0	3.0	10.0	345 ± 12.4
E2	10.0	3.0	6.5	265 ± 9.7
E3	20.0	3.0	3.0	367 ± 12.9
E4	30.0	6.0	3.0	432 ± 11.8
E5	30.0	9.0	6.5	377 ± 10.9
E6	30.0	6.0	10.0	398 ± 13.8
E7	30.0	3.0	6.5	411 ± 12.9
E8	10.0	6.0	3.0	243 ± 8.5
E9	20.0	9.0	10.0	287 ± 9.1
E10	20.0	6.0	6.5	312 ± 11.2
E11	20.0	9.0	3.0	301 ± 9.9
E12	20.0	6.0	6.5	313 ± 10.2
E13	10.0	6.0	10.0	212 ± 7.3
E14	20.0	6.0	6.5	315 ± 9.7
E15	10.0	9.0	6.5	208 ± 6.5

* Results presented as average ± SD (*n* = 3).

**Table 3:** Fit summary statistics for the globule sizes of CTD-loaded GNE.

Source	SD	Sequential *p*-Value	Lack of Fit *p*-Value	R^2^	Adjusted R^2^	Predicted R^2^	PRESS
Linear	6.86	<0.0001	0.0512	0.7659	0.9903	0.9848	1029.08
2FI	6.79	0.4131	0.0480	0.9149	0.9905	0.9759	1633.33
Quadratic	4.18	0.0506	0.1014	0.9788	0.9964	0.9806	1313.50

Abbreviations: SD, standard deviation; PRESS, predicted residual error sum of squares; 2FI, two-factor interaction.
