# Correction: Alhakamy et al. Green Nanoemulsion Stabilized by In Situ Self-Assembled Natural Oil/Native Cyclodextrin Complexes: An Eco-Friendly Approach for Enhancing Anticancer Activity of Costunolide against Lung Cancer Cells. *Pharmaceutics* 2022, *14*, 227

**DOI:** 10.3390/pharmaceutics17010107

**Published:** 2025-01-14

**Authors:** Nabil A. Alhakamy, Shaimaa M. Badr-Eldin, Osama A. A. Ahmed, Hibah M. Aldawsari, Solomon Z. Okbazghi, Mohamed A. Alfaleh, Wesam H. Abdulaal, Thikryat Neamatallah, Omar D. Al-hejaili, Usama A. Fahmy

**Affiliations:** 1 Department of Pharmaceutics, Faculty of Pharmacy, King Abdulaziz University, Jeddah 21589, Saudi Arabia; nalhakamy@kau.edu.sa (N.A.A.); oaahmed@kau.edu.sa (O.A.A.A.); haldosari@kau.edu.sa (H.M.A.); maalfaleh@kau.edu.sa (M.A.A.); al-shaery111@hotmail.com (O.D.A.-h.); uahmedkauedu.sa@kau.edu.sa (U.A.F.); 2 Center of Excellence for Drug Research and Pharmaceutical Industries, King Abdulaziz University, Jeddah 21589, Saudi Arabia; 3 Department of Pharmaceutics and Industrial Pharmacy, Cairo University, Cairo 11562, Egypt; 4 Global Analytical and Pharmaceutical Development, Alexion Pharmaceuticals, New Haven, CT 06510, USA; sokbazghi@gmail.com; 5 Vaccines and Immunotherapy Unit, King Fahd Medical Research Center, King Abdulaziz University, Jeddah 21589, Saudi Arabia; 6 Department of Biochemistry, Faculty of Science, Cancer and Mutagenesis Unit, King Fahd Medical Research Center, King Abdulaziz University, Jeddah 21589, Saudi Arabia; whabdulaal@kau.edu.sa; 7 Department of Pharmacology and Toxicology, Faculty of Pharmacy, King Abdulaziz University, Jeddah 21589, Saudi Arabia; taneamatallah@kau.edu.sa

## Error in Figure

In the original publication [1], a duplication between Figures 5 and 6 was identified. This error occurred due to an oversight during the proofreading process. The flow cytometry experiment was repeated to ensure the data’s accuracy. The corrected version of Figure 6 is presented below.

## Text Correction

In the description of “Section 2.5.3. Annexin V–FITC Apoptosis Assay”, the last three sentences are to be modified for the updated machine used as follows:

The Annexin-V FACS analyses were performed using a Cytek^®^ Northern Lights 2000 spectral flow cytometer (Cytek Biosciences, Fremont, CA, USA) and using SpectroFlo™ Software version 2.2.0.3 (Cytek Biosciences, Fremont, CA, USA).

In the description of the results in “Section 3.5. Annexin V Apoptosis Assay”, minor modification of the third sentence is requested as follows:

CTD-GNE significantly increased the percentage of early, late, and total apoptosis in A549 cells compared to the control, blank GNE, and CTD-treated cells (Figure 6).

The authors state that the scientific conclusions are unaffected. This correction was approved by the Academic Editor. The original publication has also been updated.

## Figures and Tables

**Figure 6:**
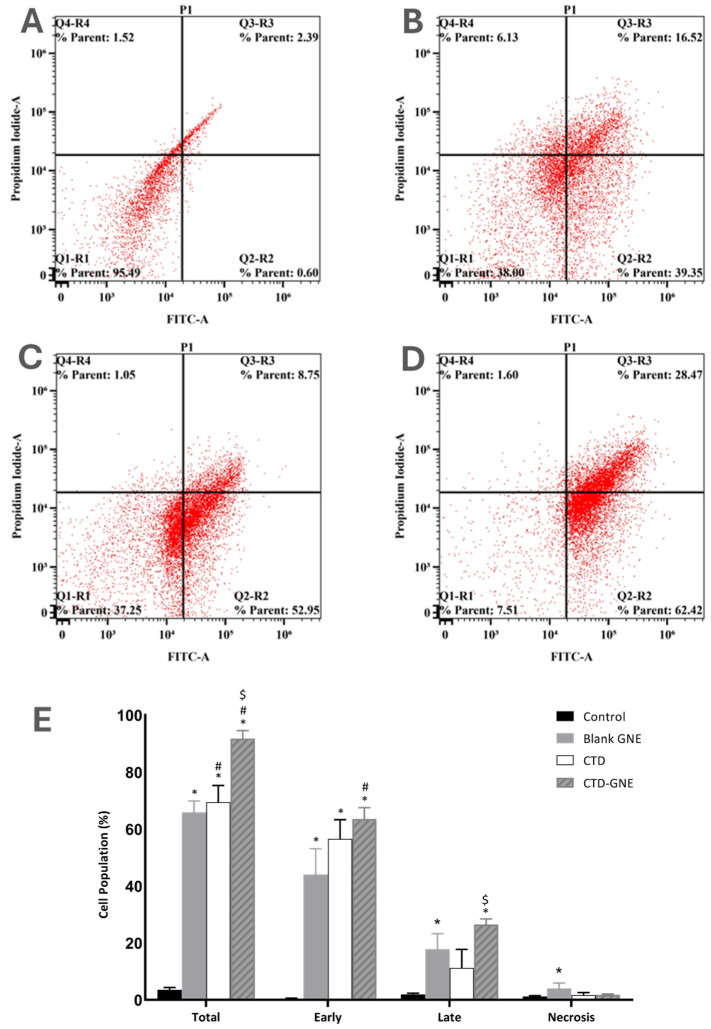
Analysis of apoptosis using the Annexin-V staining in A549 cells: (**A**) the control, (**B**) blank GNE, (**C**) CTD, (**D**) CTD-GNE and (**E**) the percentage of apoptotic or necrotic cells using flow cytometric analysis. All data are expressed as the mean ± SE of three independent experiments. * *p* < 0.05 is considered significantly different from the control, # *p* < 0.05 is considered significantly different from the blank GNE, and $ *p* < 0.05 is considered significantly different from CTD.
